# Perfectionistic Concerns and Mobile Phone Addiction of Chinese College Students: The Moderated Mediation of Academic Procrastination and Causality Orientations

**DOI:** 10.3389/fpsyg.2022.798776

**Published:** 2022-04-14

**Authors:** Guirong Liu, Xiuqin Teng, Yao Fu, Qiang Lian

**Affiliations:** Department of Teacher and Education, Qilu Normal University, Jinan, China

**Keywords:** perfectionistic concerns, academic procrastination, causality orientations, mobile phone addiction, perfectionism

## Abstract

This study aimed to investigate the effect of perfectionistic concerns (PC) on mobile phone addiction (MPA) and the mediating role of academic procrastination (AP), as well as the moderating role of causality orientations (autonomous/controlled/impersonal orientation). A cross-sectional sample of 625 Chinese college students (20.8% male, mean age = 20.47 years old) completed measures of PC, AP, causality orientations, and MPA. We analyzed the survey data using structural equation modeling (SEM) in Mplus 8.0. PC was positively related to MPA. In addition, AP partially mediated this association. The hypothesized moderating effect of autonomous orientation and controlled orientation was not supported. Impersonal orientation moderated the second stage of the mediating effect of AP on the PC–MPA link in that the mediating effect was positive when impersonal orientation was high, while the mediating effect was not significant when impersonal orientation was low. The findings confirm the importance of investigating how individual difference (i.e., PC) contributes to MPA. The implications of the findings for relieving MPA or preventing college students from developing MPA are also discussed deeply and thoroughly.

## Introduction

Smartphones have become the largest network use terminal, due to their rapid technological development and great convenience. According to the 48th Statistical Report on Internet Development in China (China Internet Network Information Center, 2021), by June 2021, the number of mobile internet users in China reached 1.007 billion, accounting for 99.6% of all internet users, of which 17.4% of internet users were in the 20–29 age group. In particular, young adults aged 18–22 years make up the largest, fastest-growing demographic of mobile phone owners and users (Chen et al., 2016). The portability and convenience of mobile phones exacerbate the risk of mobile phone addiction (MPA). As a behavioral addiction, MPA refers to excessive immersion in mobile phone activities and a strong and persistent craving and dependence on mobile phone use that led to clear social and psychological damage (Yen et al., 2009). The prevalence rate of MPA may be 10–46% in college students in many countries, and this rate is on the rise (Jun, 2016; Lian et al., 2016). MPA has negative consequences for brain structure, cognition, mental health, sleep quality, academic performance, and interpersonal relationships (Yen et al., 2009; Chen et al., 2016; Hu et al., 2017; Liu et al., 2017; Chun et al., 2018; Hong et al., 2020). In light of this, the expanding population and severe adverse effects of MPA should not be neglected.

### Perfectionistic Concerns and Mobile Phone Addiction

Perfectionism involves excessively high standards for performance, accompanied by the tendency to be overly critical evaluations of one’s behavior (Frost et al., 1990; Hewitt and Flett, 1991). Factor-analytic studies have consistently yielded two higher-order dimensions of perfectionism that cut across many perfectionism conceptualizations: perfectionistic strivings (PS) (i.e., self-oriented, personal standards, and high standards) and Perfectionistic Concerns (PC) (i.e., socially prescribed, concern over mistakes, doubts about actions, discrepancy, parental expectations, and criticism) (Stoeber, 2018). According to Stoeber and Otto (2006), PS involves setting and striving for high standards and goals for oneself; PC is characterized by harsh self-scrutiny, overly critical self-evaluations, and chronic worry about the approval of others. Stoeber and Otto pointed out that PS (an equivocal and ambivalent construct) has both positive and negative associations. For example, self-oriented perfectionism predicts high-examination performance *via* task-approach goals (Stoeber et al., 2015). Self-oriented perfectionists have a sense of personal control under low stress, whereas they exhibit high levels of depressive symptoms under high stress (Flett et al., 1995). Likewise, self-oriented perfectionism negatively predicts subjective well-being *via* low self-compassion, but positively predicts subjective well-being *via* compassion for others (Stoeber et al., 2019). Moreover, self-oriented perfectionism is related to high levels of distress *via* worry and rumination (Xie et al., 2019). In contrast to PS, research has consistently shown that PC is strongly linked to anxiety disorders, depressive symptoms, and eating disorders (Stoeber and Otto, 2006; Limburg et al., 2017). In addition, PC was the factor that distinguishes clinical forms of perfectionism from a healthy pursuit of excellence (Shafran et al., 2002; Dunkley et al., 2006). Furthermore, concern over one’s mistakes (a critical component of PC) heightens one’s susceptibility to psychological disorders (Frost and DiBartolo, 2002). Given that this study attempted to examine the relationship between PC and MPA of Chinese college students, especially the mediation process (i.e., how PC is associated with MPA) and the moderation process (i.e., when PC is associated with MPA).

Davis (2001) proposed the cognitive-behavioral model of Pathological Internet Use (PIU), which emphasizes maladaptive cognitions as proximal contributory causes of PIU. Maladaptive cognitions can be divided into two main subtypes: thoughts about oneself and thoughts about the world. Maladaptive thoughts about the self are characterized by rumination, self-doubt, low self-efficacy, and negative self-appraisal. Research showed that PC was significantly related to rumination (Abdollahi, 2019; Xie et al., 2019). Meanwhile, self-doubt, low self-efficacy, and negative self-appraisal are central features of PC (Bieling et al., 2004; Rice et al., 2012). Thus, we proposed the following hypothesis:

***Hypothesis 1***: PC is positively related to MPA.

### Academic Procrastination as a Mediator

Academic procrastination (AP) is an endemic problem among college students (Steel, 2007; Ebadi and Shakoorzadeh, 2015). Procrastination refers to the act of unnecessarily postponing or avoiding tasks to the point of experiencing personal discomfort (Solomon and Rothblum, 1984; Schraw et al., 2007). According to the temporal motivational theory (TMT), individuals with high PC worry about their mistakes, cast doubts about their actions, show a discrepancy between expectations and results, avoid disapproval by others, and excessively fear failure, which may make them more prone to postponing tasks (Steel and König, 2006). Socially prescribed perfectionism is positively related to AP for Korean 7th graders (Bong et al., 2014). Similarly, several meta-analytic studies indicate that PC was positively associated with procrastination (Steel, 2007; Xie et al., 2018). In contrast, epistemological beliefs (appropriate beliefs about the nature of knowledge and learning) predicted low levels of AP.

The significant negative aspects of AP include compromised performance, decreased learning, increased health risks, and subjective discomforts such as low academic self-efficacy, anxiety, worry, and depression (Rabin et al., 2011). The psychological decompensation hypothesis (Gao and Chen, 2006) proposes that when the normal psychological growth of an individual is hindered, the individual will turn to the internet for compensation. Moreover, pathological compensation will result in decompensation, leading to developmental deviations or interruptions and even internet addiction. For college students, AP is a deprivation of real-life and a disruption of their adaptive functions. In this case, they turn to the internet as a “refuge” to achieve a sense of accomplishment and mastery of the environment. Thus, AP represents an antecedent to MPA among college students. Given that PC is positively associated with AP and AP, in turn, is positively associated with MPA, we proposed the second hypothesis:

***Hypothesis 2***: AP mediates the positive relationship between PC and MPA.

### Causality Orientations as Moderators

Although PC may lead to AP and MPA, it is possible that not all the college students are equally influenced. Therefore, it is important to take an individual difference perspective by examining the moderating role of causality orientations. We tested the hypothesis that causality orientations would moderate the relationship between PC and MPA. Causality orientations are conceptions from causality orientations theory (COT), a mini-theory of self-determination theory (SDT) (Deci and Ryan, 1985; Ryan and Deci, 2017). There are three broad classes of behavior and motivationally relevant psychological processes: autonomous, controlled, and impersonal. Autonomy-oriented people tend to look for opportunities that provide self-determination and are actuated by choices that are based on an awareness of one’s needs and integrated goals. Control-oriented individuals are pushed by external controls (i.e., time limits, social expectations, demands from others, and material rewards) or internally controlling imperatives indicating how one “should” or “must” behave. Impersonal orientation involves people’s experiencing their behavior as being beyond their intentional control and out of their scope of competence.

Research relating to causality orientations and identity styles has revealed that autonomous orientation is positively associated with an informational identity style and negatively associated with a diffuse-avoidant style; controlled orientation is positively connected with a normative identity style and impersonal orientation predicts a diffuse-avoidant identity style (Soenens et al., 2005).

Informational identity style involves being cognitively complex, persistent, and problem focused while coping (Berzonsky, 2004). Moreover, autonomous orientation plays a significant role in the processing of threats and negative events. In contrast to other individuals, those high in autonomous orientation showed better coping when reexposed to disturbing films (Weinstein and Hodgins, 2009). Therefore, it is expected that when confronted with AP, autonomy-oriented college students will perceive procrastination as a challenging situation and adaptively cope, thus decreasing the tendency to develop MPA. Hence, autonomous orientation, as an important protective factor, may alleviate the negative effects of AP, signaling deprivation, frustration, and disruption of in-person life and adaptive functioning. In other words, the relationship between AP and MPA may be moderated by autonomous orientation.

Those with high normative identity style are less flexible and more rigid than those with high informational style. Moreover, in the face of feedback on failure, they tend to persist in a rigid ego-involved way (Koestner and Zuckerman, 1994). With regard to achievement, the controlled orientation appears to be related to the adoption of a pressured, extrinsic, and time-conscious approach to one’s activities (Deci and Ryan, 1985). Therefore, when faced with AP, control-oriented students will feel greater internal and external pressure and take actions that may be maladaptive. Control-oriented people are more likely to engage in behaviors with impaired self-discipline, such as alcohol use or gambling (Neighbors et al., 2004; Neighbors and Larimer, 2004). Specifically, when confronted with AP, individuals with high controlled orientation would be more susceptible to MPA.

The impersonal causality orientation appears to be the motivational basis of a diffuse-avoidant identity style (Soenens et al., 2005), which means that people with this orientation tend to procrastinate, deny internal conflicts, put off decisions and actions, use maladaptive coping styles, and have a high degree of neuroticism (Duriez et al., 2004). Based on this, impersonal-oriented individuals may experience more helplessness in AP and use maladaptive coping methods, such as escape, while the internet provides such a refuge. Therefore, it may be the picture that when faced with AP, impersonal-oriented students will surf the internet pathologically.

In addition, according to the diathesis-stress model (Monroe and Simons, 1991; Belsky and Pluess, 2009), negative traits can aggravate the negative consequences of adverse environments. As such, both the controlled orientation and impersonal orientation can aggravate the effect of AP on MPA. Hence, both the controlled orientation and impersonal orientation may moderate the link between AP and MPA.

Furthermore, according to previous studies on the combination of mediation and moderation models (Edwards and Lambert, 2007), if AP mediates the relationship between PC and MPA and causality orientations moderate the AP–MPA link, the mediating effect of AP would be moderated by causality orientations. It is logical to deduce that causality orientations moderate the second stage of the mediating process of AP. Thus, we proposed the third hypothesis:

***Hypothesis 3a***: Autonomous orientation moderates the second stage of the mediation model. Specifically, the mediating effect would be weaker for college students high in autonomous orientation.

***Hypothesis 3b***: Controlled orientation moderates the second stage of the mediation model. Specifically, the mediating effect would be stronger for college students high in controlled orientation.

***Hypothesis 3c***: Impersonal orientation moderates the second stage of the mediation model. Specifically, the mediating effect would be weaker for college students high in impersonal orientation.

### Present Study

**FIGURE 1:**
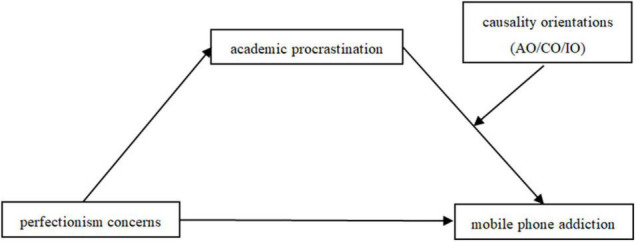
Conceptual model of AP as a mediator and causality orientations as moderators in the PC–MPA link. Note: AP, academic procrastination; AO, autonomous orientation; CO, controlled orientation; IO, impersonal orientation; PC, perfectionistic concerns; MPA, mobile phone addiction.

In sum, we aimed to examine the mediating and moderating factors underlying the relationship between PC and MPA using a moderated mediation model (Muller et al., 2005; Edwards and Lambert, 2007). The main objectives were: (1) to explore whether PC is positively associated with MPA among college students; (2) to investigate whether AP mediates the effect of PC on MPA; and (3) to determine whether the relationship between PC and MPA *via* AP varies as a function of causality orientations (autonomous/controlled/impersonal orientation). The overall moderated mediation model is given in Figure 1.

## Materials and Methods

### Participants and Procedure

The participants were 625 college students from two universities (one is a leading university with higher entrance marks, and the other is an ordinary university) in Jinan, Shandong Province in China. Of all the participants, 130 (20.8%) were male and 495 (79.2%) were female; 161 majored in education, 204 in physics, and 260 in economics, while 200 were freshmen, 237 were sophomores, and 188 were juniors. Their mean age was 20.47 (*SD* = 1.26, range = 17.83–23.58).

All the research procedures met the ethical guidelines of the American Psychological Association (APA) and were reviewed and approved by the Institutional Review Board at Qilu Normal University. We invited 697 students to voluntarily participate in the anonymous pen-and-pencil survey, which was administered at the end of each class; 93.26% (650) of the target sample consented to participate. They were assured that their responses would remain anonymous. The questionnaire took approximately 25 min to complete. Two well-trained research assistants in each classroom administered the questionnaire using scripts and a manual of procedures to standardize the data collection process. After the surveys were completed, the participants received a token gift for participation. Of the participants who consented, we excluded 25 for missing data regarding the key variables. We included a total of 625 college students in the final analysis.

### Measures

#### Demographic Features

Demographic information included gender, age, major, and academic year.

#### Perfectionistic Concerns

We used the Frost Multidimensional Perfectionism Scale (FMPS) (Frost et al., 1990) in its Chinese form [Chinese FMPS (CFMPS)] (Zi and Zhou, 2006). This was a 27-item instrument comprised of five subscales. In this study, based on the conceptual framework of Stoeber and Otto (2006) and following Rivière and Douilliez (2017), we employed three subscales as indicators of PC: concern over mistakes (e.g., “If I fail at work/school, I am a failure as a person”), doubts about actions (e.g., “Even when I do something very carefully, I often feel that it is not quite right”), and parental expectations (e.g., “My parents set very high standards for me”). The participants responded to each item on a 5-point scale (1 = strongly disagree, 5 = strongly agree). In this study, Cronbach’s α for PC was 0.85.

#### Academic Procrastination

We used the Procrastination Assessment Scale—Students (PASS) (Solomon and Rothblum, 1984) in Chinese form (Chen, 2007) to measure AP of college students. The scale assesses the prevalence of procrastination for six tasks, of which three are other-prescribed tasks, e.g., studying for an examination, writing a term paper, and performing administrative tasks. Three are self-determined tasks, i.e., participants list three goals themselves that are important to them and the ones they are carrying out. Specifically, the participants were asked to rate, on a 5-point Likert scale, the degree to which they procrastinate on the task (1 = never procrastinate; 5 = always procrastinate), the degree to which procrastination on a task is a problem for them (1 = not at all a problem; 5 = always a problem), and the degree to which they want to decrease their procrastination behavior (1 = do not want to decrease, 5 = definitely want to decrease). In this study, confirmatory factor analysis (CFA) indicated a good fit: χ^2^*/df* = 4.59, *CFI* = 0.96, *TLI* = 0.95, and *RMSEA* = 0.07. In addition, Cronbach’s α for the PASS was 0.87.

#### General Causality Orientations

We used the General Causality Orientations Scale (GCOS) (Deci and Ryan, 1985) to measure three orientations: autonomous, controlled, and impersonal. The scale demonstrated good internal consistency and construct validity (psychometric properties) in Chinese college students (Liu et al., 2012). There are 12 hypothetical vignettes, each of which describes an incident and lists three ways to respond to it. An example of a vignette is as follows: *Your company has promoted you to a position in a city far from your present location. As you think about the move you would probably: (a) feel interested in the new challenge and a little nervous at the same time* (autonomous orientation, Cronbach’s α = 0.85); *(b) feel excited about the higher status and salary involved* (controlled orientation, Cronbach’s α = 0.71); or *(c) feel stressed and anxious about the upcoming changes* (impersonal orientation, Cronbach’s α = 0.76). The participants rated how likely they would respond that way on a 7-point scale (1 = very unlikely, 7 = strongly agree). In this study, CFA indicated a good fit: χ^2^*/df* = 2.89, *CFI* = 0.97, *TLI* = 0.97, *RMSEA* = 0.05.

#### Mobile Phone Addiction

We used the MPA Index (MPAI) (Leung, 2008) to measure the MPA of college students. It is a 17-item instrument comprised of four dimensions: the inability to control a craving (e.g., “You have been told that you spend too much time on your mobile phone”), anxiety, and feeling lost (e.g., “When out of range for some time, you become preoccupied with the thought of missing a call”), withdrawal/escape (e.g., “You have used your cell phone to talk to others when you were feeling isolated”), and productivity loss (e.g., “You find yourself occupied on your cell phone when you should be doing other things, and it causes a problem”). The participants rated the items on a 5-point scale (1 = never, 5 = always). Previous studies have shown that the MPAI has good reliability and validity (psychometric properties) in Chinese adolescents and young adults (Liu et al., 2018; Hao et al., 2019). In this study, CFA indicated a good model fit: χ^2^*/df* = 3.12, *CFI* = 0.95, *TLI* = 0.95, *RMSEA* = 0.07. Cronbach’s α for the MPAI was 0.84.

### Statistical Analysis

We employed SPSS 22.0 and Mplus 8.0 for statistical analyses. First, we used *t*-tests and ANOVAs to check for possible differences in PC, AP, general causality orientations, and MPA among the demographic features, e.g., gender, academic year, and major. Second, we obtained descriptive statistics for the variables of interest using SPSS 22.0, followed by bivariate associations among the variables. Third, we conducted structural equation modeling (SEM) analysis in *Mplus* version 8.0 to test the mediating effect of AP and the moderating effect of general causality orientations in the second stage of the mediation. We tested the moderating effects of the three causality orientations one by one. Prior to analysis, we created item parcels as observed indicators using the item-to-construct balance recommended by Little et al. (2002), which we employed to measure the latent variables (PC, AP, autonomous/controlled/impersonal orientation, and MPA). We created the interaction product by multiplying the indicators of causality orientations and AP *via* the matched-pair strategy suggested by Marsh et al. (2004). We employed the bootstrapping method to test for the significance of the indirect and moderated mediation effects; the bootstrapping method produced 95% bias-corrected CIs for the indirect effect and the moderated mediation effects from 10,000 samples of the data.

### Common Method Bias

To minimize common method bias, we presented the items in a random, counterbalanced order to ensure that the questionnaire would remain anonymous. We examined common method bias using Harman’s single factor test (Podsakoff et al., 2003; Zhou and Long, 2004) and we hypothesized that there was a single common factor. The model fit the data poorly: χ^2^/*df* = 35.67, *CFI* = 0.31, *TLI* = 0.19, *RMSEA* = 0.24. Thus, common method bias did not appear to be present.

## Results

### Testing for Differences in the Demographic Variables

As shown in Table 1, the outcomes of the *t*-test revealed significant gender differences in autonomous orientation and MPA. Female students displayed significantly higher levels of autonomous orientation and MPA. The results of univariate ANOVA indicated a significant grade difference in MPA, *F*(2, 622) = 17.48, *p* < 0.001, and η^2^ = 0. 05. *Post hoc* analysis showed no significant difference between sophomores and juniors, and they all demonstrated higher levels of addiction than freshmen. In addition, there were significant major differences in MPA, *F*(2, 622) = 14.86, *p* < 0.001, and η^2^ = 0. 05. *Post hoc* analysis revealed no significant difference between college students majoring in education and those majoring in economics and they all exhibited a higher level of addiction than those majoring in physics.

**TABLE 1:** Results of demographic variables.

Variables	PC	AP	AO	CO	IO	MPA
Male	2.91 ± 0.65	2.97 ± 1.07	5.26 ± 1.03	4.99 ± 1.19	3.26 ± 1.05	2.67 ± 0.75
Female	2.81 ± 0.64	3.02 ± 0.95	5.48 ± 0.90	5.10 ± 1.01	3.29 ± 0.98	2.99 ± 0.66
*t*	0.11	0.58	–2.21*	1.02	0.39	–4.65***
Freshmen	2.85 ± 0.64	3.03 ± 1.06	5.44 ± 1.00	5.00 ± 1.14	3.19 ± 1.01	2.69 ± 0.69
Sophomores	2.88 ± 0.63	3.07 ± 0.92	5.35 ± 0.94	5.07 ± 1.01	3.35 ± 0.97	3.03 ± 0.68
Juniors	2.74 ± 0.65	2.93 ± 0.93	5.52 ± 0.84	5.18 ± 1.00	3.30 ± 1.00	3.03 ± 0.65
*F*	2.78	1.15	1.79	1.54	1.39	17.48***
Education	2.79 ± 0.62	3.02 ± 0.93	5.39 ± 0.97	4.91 ± 1.07	3.31 ± 0.98	2.96 ± 0.67
Physics	2.83 ± 0.64	2.98 ± 1.05	5.48 ± 0.94	5.10 ± 1.09	3.18 ± 1.03	2.72 ± 0.69
Economics	2.86 ± 0.66	3.04 ± 0.94	5.42 ± 0.93	5.17 ± 1.00	3.35 ± 0.96	3.06 ± 0.67
*F*	0.63	0.20	0.54	3.25	1.73	14.86***

Note: N = 625. *p < 0.05 and ^***^p < 0.001. PC, perfectionistic concerns; AP, academic procrastination; AO, autonomous orientation; CO, controlled orientation; IO, impersonal orientation; MPA, mobile phone addiction.

### Preliminary Analysis

The descriptive statistics and bivariate correlations among the variables are given in Table 2. PC was positively correlated with AP (*r* = 0.20, *p* < 0.001) and MPA (*r* = 0.32, *p* < 0.001). Moreover, AP was positively associated with MPA (*r* = 0.30, *p* < 0.001). In addition, in terms of the correlations among causality orientations and other variables, autonomous orientation was insignificantly related to AP and MPA (*ps* > 0.05), as was controlled orientation. Impersonal orientation was positively correlated with AP and MPA (*r* = 0.11, *p* < 0.01; *r* = 0.23, *p* < 0.001).

**TABLE 2:** Descriptive statistics and intercorrelations between study variables.

	1	2	3	4	5	6
1. PC	–					
2 AP	0.20***	–				
3 AO	–0.01	0.05	–			
4 CO	0.04	0.05	0.68***	–		
5 IO	0.37***	0.11**	−0.20***	−0.13**	–	
6 MPA	0.32***	0.30***	0.02	–0.02	0.23***	–
*M*	2.83	3.01	5.43	5.08	3.29	2.92
*SD*	0.64	0.97	0.93	1.05	0.99	0.69

Note: N = 625. ^**^p < 0.01 and ^***^p < 0.001. PC, perfectionistic concerns; AP, academic procrastination; AO, autonomous orientation; CO, controlled orientation; IO, impersonal orientation; MPA, mobile phone addiction.

### Testing for the Direct Effect of Perfectionistic Concerns on Mobile Phone Addiction

We conducted SEM analysis to examine the direct effect of PC on MPA. The model fit for this model was good, χ^2^*/df* = 3.67, *CFI* = 0.97, *TLI* = 0.96, *RMSEA* = 0.05. After controlling for gender, academic year, and major, PC was positively related to MPA (β = 0.43, *p* < 0.001).

### Testing for the Mediating Effect

**FIGURE 2:**
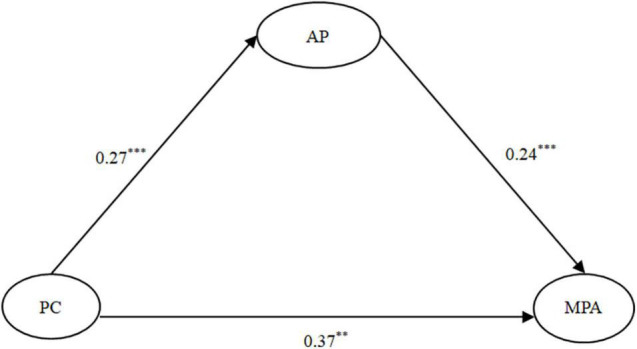
The mediating model. Note: For simplicity of presentation, control variables (gender, academic year and major) are not shown. *N* = 625. **p < 0.01 and ***p < 0.001. PC, perfectionistic concerns; AP, academic procrastination; MPA, mobile phone addiction.

To test the mediation hypothesis, we performed SEM analysis to see whether AP mediated the relationship between PC and MPA. The model fit the data well: χ^2^*/df* = 2.77, *CFI* = 0.98, *TLI* = 0.98, *RMSEA* = 0.04. As shown in Figure 2, PC was positively correlated with AP (β = 0.27, *p* < 0.001), and AP was positively related to MPA (β = 0.24, *p* < 0.001). Moreover, when AP was included, the association between PC and MPA was still significant (β = 0.37, *p* < 0.001). The indirect/mediation effect was significant, indirect effect = 0.07, *SE* = 0.02, 95% *CI* = (0.03, 0.10). The mediation effect accounted for 15.91% of the total effect of the association between PC and MPA. Namely, AP partially mediated the relationship between PC and MPA.

### Testing for the Moderated Effect of Causality Orientations

For simplicity, we tested the moderated effects of the three causality orientations one by one. In the models below, control variables, PC, AP, MPA, and the AP-causality orientations interactions were included in the model.

#### Testing for the Moderated Effect of Autonomous Orientation

**FIGURE 3:**
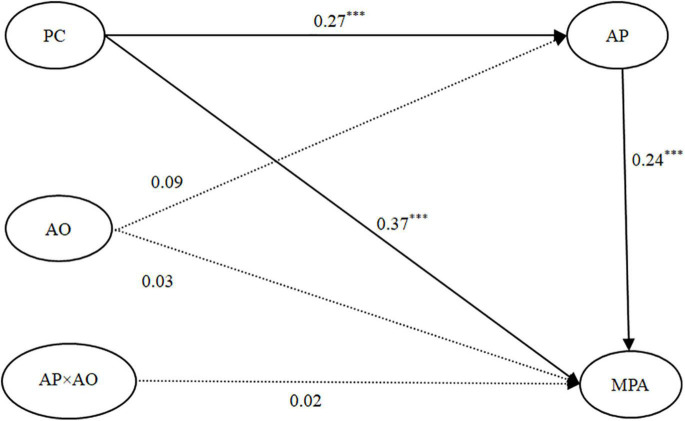
The moderated mediation model: AO as a moderating variable. Note: Standardized coefficients are presented. Control variables (gender, academic year and major) were included in the model but are not shown for simplicity. *N* = 625. ***p < 0.001. PC, perfectionistic concerns; AP, academic procrastination; AO, autonomous orientation; MPA, mobile phone addiction.

To test for the moderating effect of autonomous orientation (see Figure 3), we conducted SEM analysis and the model fit the data well: χ^2^*/df* = 2.35, *CFI* = 0.96, *TLI* = 0.96, *RMSEA* = 0.04. PC was positively correlated with AP (β = 0.27, *p* < 0.001), and AP was positively related to MPA (β = 0.24, *p* < 0.001). Moreover, the association between PC and MPA was still significant (β = 0.37, *p* < 0.001). The mediation effect was significant, indirect effect = 0.07, *SE* = 0.02, 95% *CI* = [0.03, 0.10]. The AP-autonomous orientation interaction was not significantly related to MPA (β = 0.02, *p* > 0.05). The moderating effect of autonomous orientation was not supported, and AP played a partial mediating role in the PC–MPA link.

#### Testing for the Moderated Effect of Controlled Orientation

**FIGURE 4:**
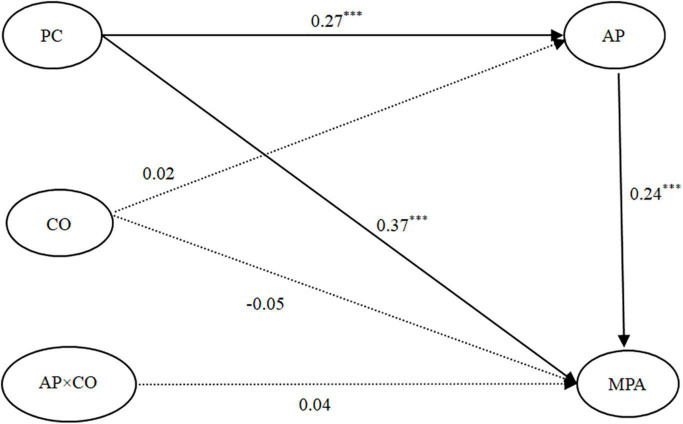
The moderated mediation model: CO as a moderating variable. Note: Standardized coefficients are presented. Control variables (gender, grade and major) were included in the model but are not presented for simplicity. *N* = 625. ***p < 0.001. PC, perfectionistic concerns; AP, academic procrastination; CO, controlled orientation; MPA, mobile phone addiction.

We performed SEM analysis to establish the moderating effect (see Figure 4), and the model fit was excellent: χ^2^*/df* = 2.14, *CFI* = 0.96, *TLI* = 0.95, *RMSEA* = 0.04. PC was positively correlated with AP (β = 0.27, *p* < 0.001), and AP was positively linked to MPA (β = 0.24, *p* < 0.001). Moreover, the association between PC and MPA was still significant (β = 0.37, *p* < 0.001). The mediation effect was significant, indirect effect = 0.06, *SE* = 0.02, 95% *CI* = (0.03, 0.10). The AP-controlled orientation interaction did not predict MPA (β = 0.04, *p* > 0.05). We did not find a moderating effect of controlled orientation, and AP played a partial mediating role in the PC–MPA link.

#### Testing for the Moderated Effect of Impersonal Orientation

**FIGURE 5:**
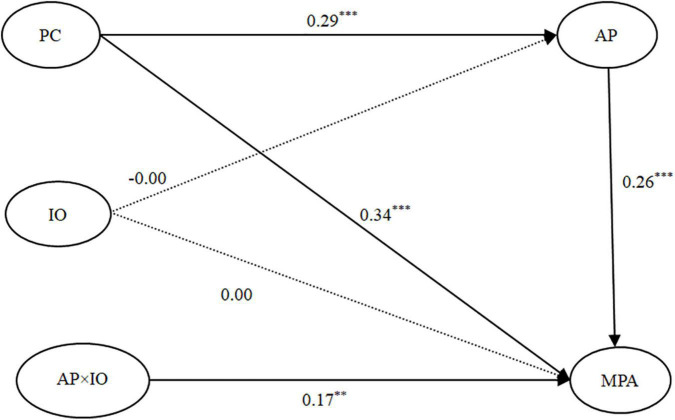
The moderated mediation model: IO as a moderating variable. Note: Standardized coefficients are presented. Control variables (gender, grade, and major) were included in the model but are not shown for simplicity. *N* = 625. **p < 0.01 and ***p < 0.001. PC, perfectionistic concerns; AP, academic procrastination; IO, impersonal orientation; MPA, mobile phone addiction.

**FIGURE 6:**
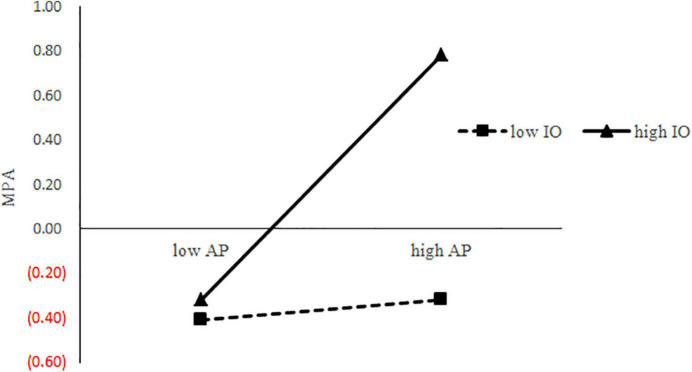
The moderating effect of IO on the relationship between AP and MPA. Conditional regressions of MPA on AP were conducted when IO was high (M + 1 SD) and low (M–1 SD). Endpoints of the lines represent MPA when AP was low or high. Note: IO, impersonal orientation; AP, academic procrastination.

We carried out SEM analysis of the moderating effect of impersonal orientation (see Figure 5) and the model fit was good: χ^2^*/df* = 2.72, *CFI* = 0.97, *TLI* = 0.96, *RMSEA* = 0.04. PC was positively correlated with AP (β = 0.29, *p* < 0.001), and AP was positively related to MPA (β = 0.26, *p* < 0.001). Moreover, the relationship between PC and MPA was still significant (β = 0.34, *p* < 0.001). The mediation effect was significant, indirect effect = 0.08, *SE* = 0.02, 95% *CI* = (0.03, 0.012). The interaction of AP and impersonal orientation was positively correlated with MPA (β = 0.17, *p* < 0.01). This indicated that impersonal orientation moderated the second stage of the mediation. To better understand the moderating effect of impersonal orientation, we measured the interaction effect using the procedure described by Stone and Hollenbeck (1989). As shown in Figure 6, AP was positively related to MPA when impersonal orientation was high (β*_*simple*_* = 0.47, *p* < 0.001), but not to MPA when impersonal orientation was low (β*_*simple*_* = 0.05, *p* > 0.05).

According to Edwards and Lambert (2007), when the mediation is moderated, it is necessary to test whether the mediating effects vary significantly in accordance with the change in the moderating variable. Specifically, PC had a positive and indirect relationship with MPA when impersonal orientation was high (β = 0.30, *p* < 0.001), 95% *CI* = (0.11, 0.57) while PC was not significantly related to MPA when impersonal orientation was low (β = 0.04, *p* > 0.05) 95% *CI* = (–0.18, 0.014).

## Discussion

Numerous studies draw attention to the negative effects of MPA and have garnered considerable empirical support (Hu et al., 2017; Hong et al., 2020). Thus, it is important to explore factors that influence the MPA of college students and to develop targeted prevention and intervention approaches. This study extends the previous literature by exploring the relationship between PC and MPA, as well as the underlying mediating and moderating mechanisms. We set up a moderated mediation model to establish whether AP mediated the relationship between PC and MPA, and whether causality orientations moderated the relationship between PC and MPA. The results show that the effect of PC on MPA can be partially explained by AP. Moreover, this indirect link was moderated by impersonal orientation in the second stage of the mediation process, with the effects being significantly positive for students high in impersonal orientation and insignificant for those low in impersonal orientation. These findings contribute to a better understanding of how and when PC is associated with MPA.

### Perfectionistic Concerns, Academic Procrastination, and Mobile Phone Addiction

This study indicates that PC was positively associated with MPA, signaling that PC is an important diathesis of MPA and that students with high PC are more susceptible to MPA. This result coincides with the cognitive-behavioral model of PIU described by Davis (2001). The model underlines the vital role of maladaptive cognitions in contributing to PIU. This finding has important theoretical implications in that it expands the scope of the model to MPA.

In addition, AP partially mediated the PC–MPA link. For the first stage of the mediation process (i.e., the association between PC and AP), our finding is consistent with TMT, which proposes that individuals with high PC would presumably postpone tasks because of worries, doubts, and fears. For the second stage (i.e., the AP–MPA link), the results coincide with the psychological decompensation hypothesis proposed by Gao and Chen (2006). Similarly, Suler (1999) and Kardefelt-Winther (2014) proposed that internet addiction or PIU often originates from unsatisfaction of people with the offline world (e.g., in-person life and adaptive functioning have been deprived, frustrated, or disrupted). Since AP represents a deprivation of academic life and produces motivation to compensate, college students turn to the internet to regain a sense of accomplishment and mastery of the environment. It is noteworthy that in addition to the indirect effect of PC on MPA, the direct effect of PC on MPA is still significant. This outcome underlines the key role of PC in predicting MPA.

The results confirm our hypothesis and add to the limited literature on AP as an explanatory factor for how PC could contribute to MPA. Although previous studies have shown associations between PC and anxiety, depressive symptoms, eating disorders, and exercise addiction/dependence (Bieling et al., 2004; Di Schiena et al., 2012; Costa et al., 2016; Limburg et al., 2017; Rivière and Douilliez, 2017; Çakın et al., 2021), little research has explored the relationship between PC and MPA. Fewer studies have revealed the mediating mechanism between PC and MPA. This study goes beyond past research by documenting how PC could increase MPA, which could be of great help in the prevention of and interventions for MPA in today’s digital era.

### Moderating Role of Causality Orientations

Our third objective was to explore the moderating role of causality orientations in the indirect PC–MPA link *via* AP. The moderating role of impersonal orientation was confirmed, while the moderating roles of autonomous orientation and controlled orientation were not supported. As implied by Figure 6, when the level of impersonal orientation is high, the mediating effect is positive, and when impersonal orientation is low, the mediating effect is non-significant. In other words, for college students with high impersonal orientation, PC predicts more AP, which in turn leads to more MPA. Presumably, college students with PC may be more likely to procrastinate. AP entails setbacks or even failures, especially for those high in impersonal orientation. Since impersonal orientation involves negative self-evaluations and helplessness, AP “verifies” the identification of their inferiority. In addition, as a result, they turn to the internet for “asylum.”

This finding is consistent with prior research (Soenens et al., 2005) indicating that impersonal orientation is related to a diffuse-avoidant identity style, and when faced with difficulties and frustrations, students with high impersonal orientation are more likely to escape simply. It also provides further evidence for the diathesis-stress model (Monroe and Simons, 1991; Belsky and Pluess, 2009). As a maladaptive causality orientation, impersonal orientation can exacerbate or, in its relative absence, lessen the adverse consequences of AP, e.g., MPA.

Unlike we assumed, neither autonomous orientation nor controlled orientation moderated the PC–MPA link *via* AP. The non-significant findings may be explained by the interpretations presented below. First, because people high in autonomous orientation base their actions on their personal interests and perceive their behavior as being freely chosen, they will perceive AP as a challenge if they are interested in academic tasks as predicted based on SDT. However, if they are not interested in such tasks, they will probably not take action due to external stress. Second, unlike senior high schools where AP is the almost sole criterion of excellence, the criteria in universities are multiple and latent, and academic performance is just one aspect. Most likely, AP failed to trigger the control-oriented concerns of students over external contingencies.

## Limitations and Implications

The present study advances our understanding of the MPA of college students and provides empirical evidence for the cognitive-behavioral model of PIU discussed by Davis (2001). Some limitations, however, must be considered when interpreting the findings. First, doubt about the direction of causality may arise due to the cross-sectional nature of the data. We constructed a moderated mediation model based on the psychological decompensation hypothesis. However, it is also possible that college students who use mobile phones pathologically have a tendency to procrastinate in their academic tasks (Dong et al., 2016; Hawi and Samaha, 2016). Thus, longitudinal and/or experimental designs are needed to establish causal relations and ascertain the causal direction of the results. Second, the data were based on the self-reports of college students, which may lead to the common method bias. Although the questionnaire has been validated by previous research, future studies would benefit from using multiple informants to collect data, such as from teachers and peers. Third, our results highlight the importance of PC in the development of MPA among college students, and future research could examine the potential impact of other factors, such as life events (Flett et al., 1995) and perceived stress (Eitle et al., 2013; Jun and Choi, 2015). Finally, the moderating roles of autonomous orientation and controlled orientation were not supported. The findings were interpreted using aspects such as the characteristics of autonomous orientation and controlled orientation, and the multiple criteria of excellence for universities, which requires further empirical study for verification.

Despite these limitations, our findings have important practical implications. First, this study goes beyond the current literature by exploring the relationship between PC and MPA and indicates that PC is a significant risk factor for MPA. Thus, it is important to pay attention to college students high in PC to prevent MPA. Mindfulness training (Sollie et al., 2017) and cognitive control (Hoorelbeke et al., 2016) may reduce the rumination of people (a construct significantly related to PC) in the face of stress. Second, given that AP is a significant risk factor linking PC and MPA, reducing AP may be of importance to prevent college students from developing MPA. Solomon and Rothblum (1984) showed that fear of failure and aversiveness to a task are the two strongest predictors of AP. Therefore, it makes sense for educators to take measures to strengthen the self-efficacy of college students and nurture their interests in their academic tasks. Third, this study demonstrates the risk-amplifying role of impersonal orientation in the second stage of the mediation process. It is crucial for educators to help college students endorse an internal locus of control and perform attribution training.

## Conclusion

Our purpose was to investigate MPA within the framework of the cognitive-behavioral model, psychological decompensation hypothesis, and SDT. A moderated mediation model of MPA fit the data well. The research emphasizes the preponderant role of PC as predisposed vulnerability and the role of AP as reinforcement for MPA. It also draws attention to the importance of considering the causality orientations of individuals when studying MPA. The results contribute to the literature by advancing our understanding of MPA and providing vital practical implications.

## Data Availability Statement

The raw data supporting the conclusions of this article will be made available by the authors, without undue reservation.

## Ethics Statement

The studies involving human participants were reviewed and approved by the Qilu Normal University Institutional Review Board. The patients/participants provided their written informed consent to participate in this study.

## Author Contributions

GL: substantial contributions to the design of the work, data collection, data analysis, and writing. XT: substantial contributions to the design of the work, data collection, data analysis, and revising the work critically. YF and QL: substantial contributions to the writing and revising the work critically. All authors contributed to the article and approved the submitted version.

## Conflict of Interest

The authors declare that the research was conducted in the absence of any commercial or financial relationships that could be construed as a potential conflict of interest.

## Publisher’s Note

All claims expressed in this article are solely those of the authors and do not necessarily represent those of their affiliated organizations, or those of the publisher, the editors and the reviewers. Any product that may be evaluated in this article, or claim that may be made by its manufacturer, is not guaranteed or endorsed by the publisher.
